# A Single Dose, Randomized, Open‐Label, Cross‐Over Bioequivalence Study of Budesonide Pressurized Metered‐Dose Inhaler in Healthy Chinese Subjects

**DOI:** 10.1002/prp2.70197

**Published:** 2025-11-29

**Authors:** Kai Huang, Shaohong Yin, Weiguo Huang, Lairong Ding, Wei Qin, Zhenzhong Qian, Ying Ding, Linling Que, Yunfei Shi, Jinxia Gao, Yi Zhao

**Affiliations:** ^1^ Drug Clinical Trial Institution, The Affiliated Wuxi People's Hospital of Nanjing Medical University, Wuxi People's Hospital, Wuxi Medical Center, Nanjing Medical University Wuxi China; ^2^ Lunan Better Pharmaceutical Co. LTD Shandong China

**Keywords:** bioequivalence, budesonide, pharmacokinetics, pressurized metered dose inhaler

## Abstract

Budesonide is a first‐line inhaled glucocorticoid (ICS) for asthma treatment in adults and children. The purpose of this study was to compare the pharmacokinetics and assess the bioequivalence between two budesonide pressurized metered‐dose inhalers (pMDIs, 200 μg/actuation × 1 actuation) in healthy Chinese subjects. The study was conducted in 32 healthy Chinese subjects using a single‐center, randomized, open‐label, four‐period and crossover design with a 3‐day washout between periods. Blood samples were collected up to 16 h post‐dose. Plasma concentrations of budesonide were quantified using liquid chromatography–tandem mass spectrometry (LC–MS/MS). Reference‐scaled average bioequivalence (RSABE) or average bioequivalence (ABE) method was applied to evaluate the bioequivalence, on the basis of the within‐subject standard deviation (*S*_WR_) of the reference product (Budiair), and the safety was also assessed. Eventually, 31 subjects completed this study. For the maximum concentration (*C*_max_) (within‐subject standard deviation, *S*_WR_ ≥ 0.294), the RSABE method showed a geometric mean ratio (GMR) of 97.13% with a 95% upper confidence bound of < 0. For the area under plasma concentration‐time curve from time zero to the last measurable concentration (AUC_0−t_) and the area under plasma concentration‐time curve extrapolated to infinity (AUC_0−∞_) (*S*_WR_ < 0.294), ABE yielded GMRs of 104.81% and 104.61%, with 90% confidence intervals (CIs) of 99.98%–109.86% and 99.81%–109.63%, respectively. All adverse events (AEs) were mild to moderate and transient, with no serious adverse events (SAEs) reported. The two budesonide pMDIs (200 μg) were bioequivalent and well tolerated in healthy Chinese subjects.

**Trial Registration:** Chinese Clinical Trial Registry, Registration No. CTR20244600; ClinicalTrials.gov identifier: NCT06924190

**Abbreviations**
ABE: average bioequivalence
AE: adverse events
AUC_0−∞_
: area under plasma concentration‐time curve extrapolated to infinity
AUC_0−T_
: area under plasma concentration‐time curve from time zero to the last measurable concentration
BES: bioequivalence set
BMI: body mass index
CI: confidence intervals
*C*_max_
: maximum concentration
CV: coefficient of variation
CV_W_
: within‐subject coefficient of variation
ECG: electrocardiograph
FAS: full analysis set
FDA: food and drug administration
GMR: geometric mean ratio
ICS: inhaled glucocorticoids
IS: internal standard
LC–MS/MS: liquid chromatography–tandem mass spectrometry
Meddra: medical dictionary for regulatory activities
MRM: multiple‐reaction‐monitoring
NCI‐CTCAE: National Cancer Institute‐Common Terminology Criteria for Adverse Events
NMPA: National Medical Products Administration
Oips: orally inhaled products
PK: pharmacokinetics
PKCS: pharmacokinetics concentration set
PKPS: pharmacokinetics parameter set
Pmdi: pressurized metered‐dose inhalers
RSABE: reference‐scaled average bioequivalence
SAE: serious adverse events
SS: safety analysis set
*S*_WR_
: within‐subject standard deviation
*T*_1/2_
: terminal elimination half‐life

## 1 Introduction

Asthma, a common chronic respiratory disease, is recognized as a major global public health problem [1]. Currently, over 300 million people are affected worldwide, with a continuously rising prevalence [2]. Asthma is characterized by chronic airway inflammation and hyperresponsiveness, manifesting as wheeze, cough, chest tightness, and/or dyspnea [3, 4]. It imposes significant burdens on both individual health and the socioeconomic system [5]. Therefore, how to effectively treat and manage asthma has become an important goal for the prevention of asthma in recent years.

Inhaled glucocorticoids (ICSs) are the first‐line treatment for asthma because of their potent anti‐inflammatory effects and favorable safety profiles [6]. Budesonide, one of the most widely prescribed ICSs, is used in both adults and pediatric patients owing to its potent anti‐inflammatory activity, high airway selectivity, low systemic bioavailability, and minimal side side effects [7, 8]. Following inhalation, budesonide is rapidly absorbed into the lungs because of its moderate lipophilicity, approximately 15% deposits in the airways from a pressurized metered dose inhaler (pMDI), only 10% of ingested budesonide reaches the systemic circulation, with excretion via the urine and feces [9, 10]. Additionally, budesonide is extensively metabolized by cytochrome P450 (CYP) isoenzyme 3A4 into two inactive metabolites, 16α‐hydroxyprednisolone and 6β‐hydroxybudesonide, which exhibit low glucocorticoid receptor affinity [11]. CYP3A4 inhibitors may potentially impact the metabolism of budesonide [12].

Budesonide pMDI (Budiair) is manufactured by Chiesi S.A.S (Colombes, France). A new generic budesonide pMDI has been developed by Lunan Better Pharmaceutical Co. LTD (Shandong, China). To comply with China's National Medical Products Administration (NMPA) regulations for generic inhaled products approval, the bioequivalence study was required [13]. Despite variations in international bioequivalence guidelines, pharmacokinetics (PK) studies remain the gold standard for demonstrating the bioequivalence between test and reference products in vivo [14]. The study aimed to evaluate the bioequivalence and safety of a generic budesonide pMDI compared with the marketed budesonide pMDI (Budiair) in healthy Chinese subjects under fasting conditions.

## 2 Methods

This study was conducted at the phase I center of Wuxi People's Hospital between December 2024 and January 2025 in compliance with the International Council for Harmonisation Good Clinical Practice, the principle of the Declaration of Helsinki and the applicable local regulatory requirements. The study protocol and informed consent form were approved by the Independent Ethics Committee of Wuxi People's Hospital (Ethics Approval No: 2024LLPJ‐I‐123).

### 2.1 Subjects

Healthy Chinese males and females aged 18–60 years with a body mass index (BMI) of 19.0–26.0 kg/m^2^ were eligible for inclusion. Subjects were determined to be healthy on the basis of vital signs, physical examination, medical history, laboratory tests (including blood chemistry, hematology, and urinalysis), virological examinations (human immunodeficiency virus antibody, hepatitis B antigen, hepatitis C antibody, and *treponema pallidum* antibody), chest X‐ray, electrocardiograph (ECG), pulmonary function test, urine nicotine test, alcohol breath analysis, and urine drug screening test within 2 weeks prior to the study. Subjects were excluded if they were allergic to any biologic agents, took any medicine within 30 days prior to the study or attempted to become pregnant or breastfeed. More importantly, subjects should be able to use the metered‐dose inhaler correctly.

Eligible subjects were admitted to the Phase I Clinical Trial Center. They were required to fast for at least 10 h before dosing and maintain a light diet during the study. Consumption of any food or beverages containing methylxanthines (e.g., coffee, tea, cola, and cocoa), alcohol, or grapefruit, as well as the use of any other forbidden medications, was strictly forbidden.

All subjects were informed about the objectives, procedures, and potential risks of the study before signing the written informed consent. Subjects retained the right to withdraw from the study at any time without providing justification or incurring penalties.

### 2.2 Clinical Study Design

This was a single‐center, randomized, open‐label, four‐period, crossover study. 32 healthy Chinese subjects were randomly assigned to the TRTR group (*n* = 16) and RTRT group (*n* = 16) in a 1:1 ratio according to the random table before dosing.

**FIGURE 1:**
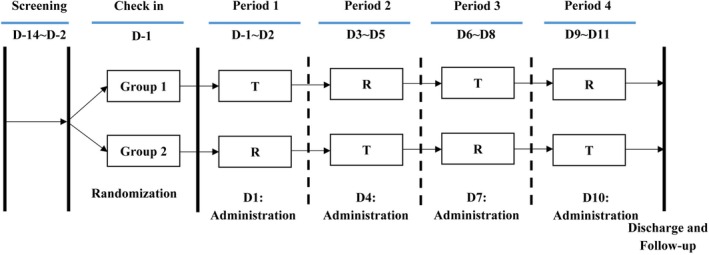
Study design.

Subjects received the test product (Budesonide pMDI, 200 μg per actuation × 1 actuation, lot no. 558240501; Lunan Better Pharmaceutical Co. LTD., Shandong, China) or reference product (Budiair, 200 μg per actuation × 1 puff, lot no. 1186054; Chiesi S.A.S, Colombes, France) under fasting conditions in each period (Figure 1), with a 3‐day washout between periods.

For standardized administration, subjects inhaled slowly through their mouths while actuating the budesonide pMDI, immediately held their breath for 10 s, and then exhaled slowly. They were instructed to rinse their mouths thoroughly with water post‐administration. All subjects remained fasting for 4 h post‐dose. With water restriction 1 h before and after dosing.

The pharmacokinetic (PK) samples (5.0 mL) were collected in K_2−_EDTA vacuum tubes at 0 (pre‐dose) and 0.03, 0.07, 0.10, 0.13, 0.17, 0.20, 0.25, 0.33, 0.42, 0.50, 0.75, 1, 2, 4, 6, 8, 10, 12, and 16 h after administration. Samples were centrifuged at 1700× *g* for 10 min at 4°C and then divided into two portions (test and backup) and stored at ≤ −60°C until analysis.

The plasma budesonide concentrations were quantified by a validated liquid chromatography–tandem mass spectrometry (LC–MS/MS) method [11]. Budesonide (purity: 98.27%) and budesonide‐d8 (internal standard, IS; purity: 98.70%) were purchased from CATO Research Chemical Inc. (Guangdong, China) and TLC Pharmaceutical Standards Ltd. (Ontario, Canada), respectively. Budesonide was extracted from the plasma sample using liquid–liquid extraction with methyl tertiary butyl ether, and a 20.0 μL extracted sample was injected into the LC–MS/MS with gradient elution. Budesonide and budesonide‐d8 were detected by the AB SCIEX Triple Quad 7500 system in multiple‐reaction‐monitoring (MRM) with negative‐ion electrospray ionization. The mass transition ion pairs were m/z 489.2 → 357.2 for budesonide and m/z 497.2 → 357.2 for budesonide‐d8 (IS). The calibration curve of budesonide was validated over the concentration range of 4.0–3000 pg/mL with the lower limit of quantification of 4.0 pg/mL. The inter‐ and intra‐batch precisions were less than 10% and the accuracies were all within ±15%. Budesonide in the extracted plasma samples was stable for 285 h at 2°C–8°C and 25 h at 20°C–30°C, and budesonide in plasma was stable for 57 days at −80°C.

### 2.3 Safety Assessment

Safety was assessed by clinical observation and spontaneous reporting of adverse events (AEs). Clinical laboratory tests (blood chemistry, hematology, and urinalysis), ECG, physical examination, and vital signs were also performed at the end of the study. The seriousness and severity of AEs and the relationship to the investigational products were recorded and reported faithfully. All AEs were coded according to the Medical Dictionary for Regulatory Activities (MedDRA) (version 27.1), with the seriousness and severity recorded according to the National Cancer Institute–Common Terminology Criteria for Adverse Events (NCI‐CTCAE) (version 5.0).

### 2.4 Statistical Analysis

The calculation of sample size for a bioequivalence study is mainly based on the power value, the intra‐individual coefficient of variation (CV%), and the test/reference ratio [15]. The intra‐subject CV% for the maximum concentration (*C*_max_) of budesonide was assumed to be 30% according to the previous literature [16]. The geometric mean ratio (GMR) of the test/reference for *C*_max_ was set at 95% to achieve 90% power (1‐β) at the significance level (two‐sided *α* = 5%). The initial sample size was 26, calculated by PASS software (version 22). Considering the 20% drop‐out rate, the final sample size was determined to be 32.

PK analysis was performed by the non‐compartmental method with WinNonlin software (version 8.3). The main PK parameters included *C*_max_, the time to maximum concentration (*T*_max_), the area under the plasma concentration‐time curve from time zero to the last measurable concentration (AUC_0−*t*_), the area under the plasma concentration‐time curve extrapolated to infinity (AUC_0−*∞*_), and the terminal elimination half‐life (*t*_1/2_).

The reference‐scaled average bioequivalence (RSABE) and average bioequivalence (ABE) methods were used for bioequivalence evaluation, with the within‐subject standard deviation (*S*_WR_) of the reference product calculated first. The RSABE method was applied if *S*_WR_ ≥ 0.294 (the within‐subject coefficient of variation, CV_W_% ≥ 30%) for any primary PK parameters (*C*_max_, AUC_0−*t*_ or AUC_0−∞_). Bioequivalence was concluded when the geometric mean ratios (GMRs) for *C*_max_, AUC_0−*t*_, and AUC_0−*∞*_ fell within 80%–125%, and the 95% upper confidence bound for (*Y*_T_ − *Y*_R_)^2^—*θ* × (*S*_WR_)^2^ was ≤ 0. *Y*_R_ and *Y*_T_ are the mean values of the natural log‐transformed AUC or *C*_max_ for test and reference products, respectively. *θ* = (ln (1.25)^2^/*σ*_W0_)^2^ is the bioequivalence limit, and *σ*_W0_ = 0.25 is the regulatory constant specified in guidance. The ABE method was used if *S*_WR_ < 0.294 (CV_W_% < 30%). Bioequivalence was concluded when the 90% confidence intervals (CIs) for the GMR of *C*_max_, AUC_0−*t*_, and AUC_0−∞_ were all within 80%–125% [17].

The normality of PK parameters was assessed using the Shapiro–Wilk test. Data were analyzed using the paired *t*‐test for normally distributed parameters and the Wilcoxon signed‐rank test for non‐normally distributed parameters. All statistical analyses were performed using SAS software (version 9.4). *p* < 0.05 was considered statistically significant.

## 3 Results

### 3.1 Demographic Data of Subjects

**FIGURE 2:**
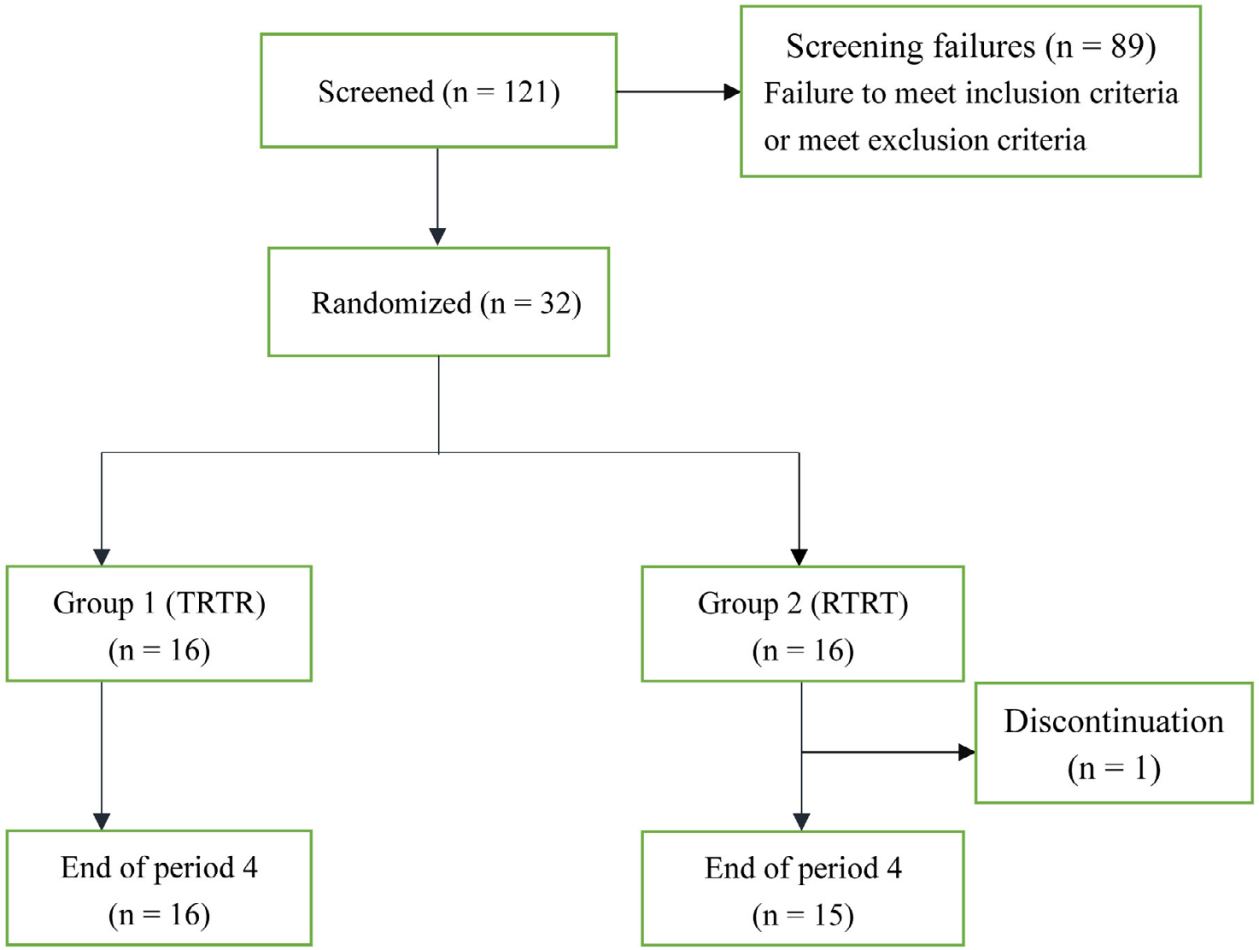
Study flow diagram.

A total of 121 subjects were screened, of which 32 subjects (28 males and four females) met the inclusion criteria and were enrolled. Ultimately, 31 subjects completed this study, whereas one subject discontinued after the first treatment period because of an AE (fever) (Figure 2). The mean age of subjects was 29.53 years (range: 18–39 years), mean body weight was 65.77 kg (range: 54.1–81.5 kg), mean height was 169.74 cm (range: 151.1–181.6 cm), and the mean BMI was 22.80 kg/m^2^ (range: 19.8–25.9 kg/m^2^) (Table 1).

**TABLE 1:** The demographic data of enrolled subjects.

	Age (y)	Weight (kg)	Height (cm)	BMI (kg/m^2^)
Mean ± SD	29.5 ± 5.7	65.8 ± 7.0	169.7 ± 7.4	22.8 ± 1.6
Median	31.0	65.8	170.4	22.6
Minimum	18.0	54.1	151.1	19.8
Maximum	39.0	81.5	181.6	25.9

Abbreviations: BMI, body mass index; SD, standard deviation.

All subjects were included in the full analysis set (FAS), safety analysis set (SS), pharmacokinetics concentration set (PKCS), pharmacokinetics parameter set (PKPS), and bioequivalence set (BES).

### 3.2 Pharmacokinetic Analyses

**FIGURE 3:**
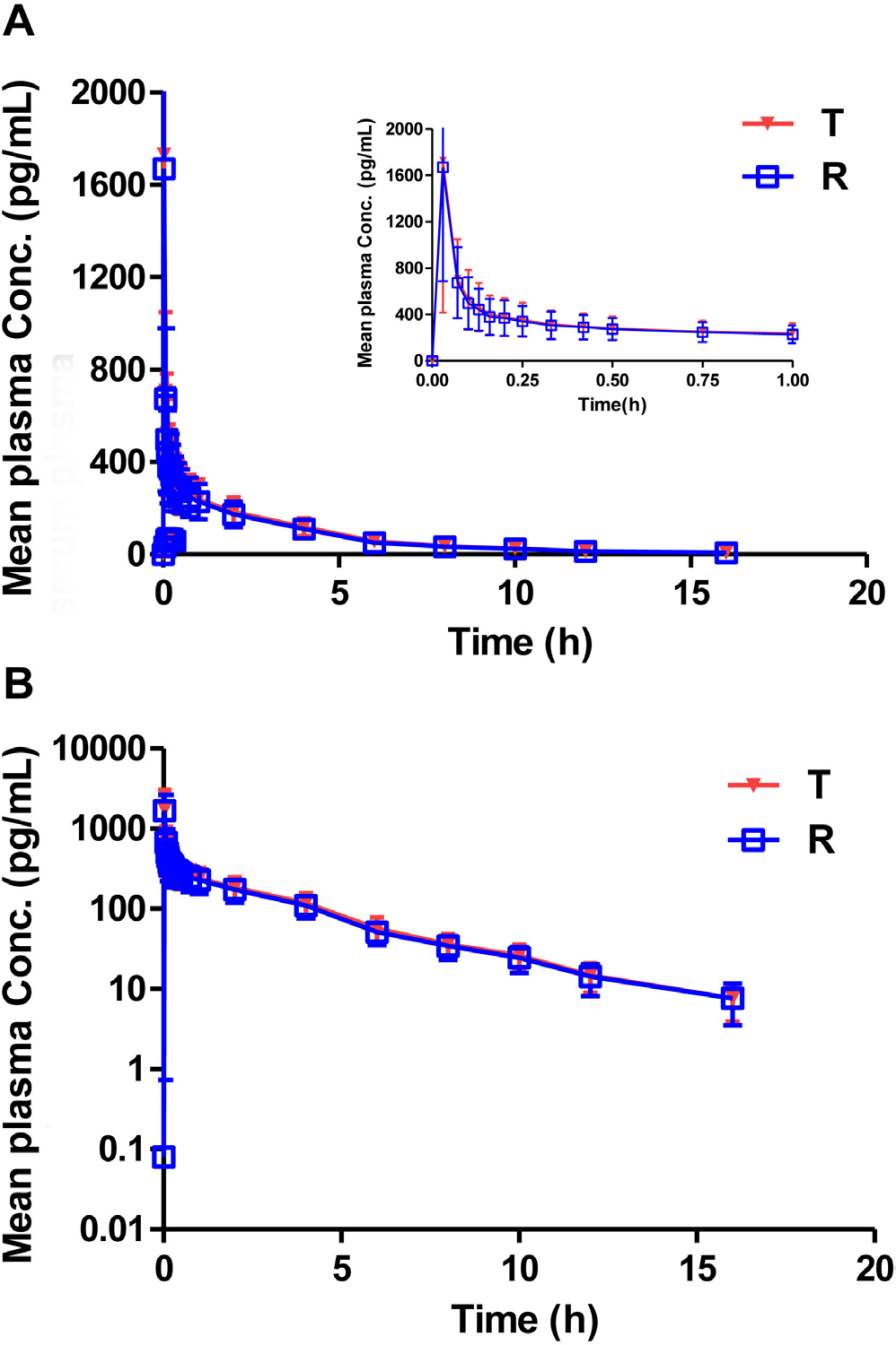
Mean plasma concentration‐time curves (A) and semilogarithmic curves (B) of the test and reference products of budesonide pMDI in Chinese healthy subjects (mean ± standard deviation [SD]). *N* = 62 for the test product and *N* = 63 for the reference product; 31 subjects successfully completed the study, whereas only 1 subject completed the first period and withdrew from the study.

PK parameters of the test product (budesonide pMDI) and the reference product (Budiair) are summarized in Table 2. *C*_max_ was 1782.11 ± 1266.01 and 1688.02 ± 960.69 pg/mL, AUC_0–t_ was 1286.63 ± 427.53 and 1219.33 ± 376.96 h pg/mL, AUC_0–∞_ was 1324.51 ± 437.15 and 1259.22 ± 395.27 h pg/mL, and *t*_1/2_ was 3.13 ± 0.59 and 3.26 ± 0.69 h. No significant differences were observed in any of the above measured PK parameters (*p* > 0.05). The mean plasma concentration‐time curves of budesonide were similar and overlapping after inhalation of 200 μg of budesonide pMDI or Budiair in healthy Chinese subjects (Figure 3).

**TABLE 2:** The primary pharmacokinetic parameters after inhalation of 200 μg of Budesonide pMDI in healthy Chinese subjects.

Parameter	Test (*n* = 31)	Reference (*n* = 32)	*P* values
*C*_max_ (pg/mL)	1782.11 ± 1266.01	1688.02 ± 960.69	0.5271
AUC_0–t_ (h pg/mL)	1286.63 ± 427.53	1219.33 ± 376.96	0.1171
AUC_0–∞_ (h pg/mL)	1324.51 ± 437.15	1259.22 ± 395.27	0.1392
*T*_max_ (h)	0.03 (0.03, 2.00)	0.03 (0.03, 0.13)	0.1198
*t*_1/2_ (h)	3.13 ± 0.59	3.26 ± 0.69	0.0919

*Note:* No statistically significant differences were found between the above parameters (*p* > 0.05). *T*_max_ represented with Median (Minimum, Maximum) and others represented with mean ± standard deviation.

Abbreviations: AUC_0–∞_, the area under plasma concentration–time curve extrapolated to infinity (AUC_0–∞_); AUC_0–t_, the area under plasma concentration–time curve from time zero to the last measurable concentration; *C*_max_, the maximum concentration; *t*_1/2_, the terminal elimination half‐life; *T*_max_, the time to maximum concentration.

As shown in Table 3, the *C*_max_ of Budiair had a *S*_WR_ of 0.4229, which meant CV_W_% of 44.26% was greater than 30%. Therefore, the RSABE method was used to evaluate the bioequivalence of *C*_max_, and the 95% upper confidence bound was −0.0922. The GMR for *C*_max_ between the test and reference products was 97.13%, which fell within 80.00%–125.00%. For AUC_0−*t*_ and AUC_0−∞_ of Budiair, the *S*_WR_ was less than 0.294, which meant CV_W_% was less than 30%; the ABE method was used. The GMRs and their 90% CIs for AUC_0−*t*_ and AUC_0−∞_ were 104.81% (99.98%–109.86%) and 104.61% (99.81%–109.63%), respectively, both falling within the range of 80.00%–125.00%.

**TABLE 3:** Bioequivalence Evaluation of test and reference products of budesonide pMDI in healthy Chinese subjects.

Parameter	GMR (T/R)	90% CI	Power	Intra‐subject CV	*S*_WR_	Upper 95% CI	Evaluation method
Cmax (pg/mL)	97.13%	84.71%–111.36%	99.54%	44.26%	0.4229	−0.0922	RSABE
AUC_0–*t*_ (h pg/mL)	104.81%	99.98%–109.86%	> 99.99%	16.72%	0.1661	—	ABE
AUC_0–∞_ (h pg/mL)	104.61%	99.81%–109.63%	> 99.99%	16.74%	0.1663	—	ABE

Abbreviations: AUC_0–∞_, the area under plasma concentration‐time curve extrapolated to infinity; AUC_0–t_, the area under plasma concentration‐time curve from time zero to the last measurable concentration; CI, confidence interval; *C*_max_, the maximum concentration; CV, the intra‐individual coefficient of variation; GMR, the geometric mean ratio; R, reference; *S*_WR_, the within‐subject standard deviation; T, test; *t*_1/2_, the terminal elimination half‐life; *T*_max_, the time to maximum concentration.

### 3.3 Safety Analysis

In our study, four subjects (12.5%) experienced five AEs. Table 4 details the AEs reported in healthy Chinese subjects following inhalation of either the test or reference product. Both products demonstrated acceptable tolerability profiles in 32 enrolled healthy Chinese subjects who were all included in SS.

**TABLE 4:** Summary of adverse events reported in healthy Chinese subjects inhaling 200 μg of budesonide pMDI.

AEs	Test (*n* = 31)	Reference (*n* = 32)	Severity	Overall (*n* = 32)
	*N* (%)	*N* (%)		
Blood glucose decreased	1 (3.2)	1 (3.1)	Mild	2 (6.3)
Aspartate aminotransferase increased	1 (3.2)	0	Mild	1 (3.1)
Alanine aminotransferase increased	1 (3.2)	0	Mild	1 (3.1)
Fever	0	1 (3.1)	Moderate	1 (3.1)

Abbreviations: AEs, adverse events; *N* (%), number (%) of subjects reporting adverse events.

Three AEs occurred in two subjects (6.5%) after inhalation of the test product during the end‐of‐study safety examinations, including blood glucose decrease (*n* = 1, 3.2%), AST increase (*n* = 1, 3.2%), and ALT increase (*n* = 1, 3.2%). The AST and ALT elevations were assessed as possibly drug‐related, whereas blood glucose decrease was considered unlikely related. All AEs were mild and resolved spontaneously within 1 week.

During the end‐of‐study safety examinations, one subject experienced one AE, blood glucose decreased (*n* = 1, 3.1%), following inhalation of the reference product. It was deemed unlikely to be drug‐related and mild in severity, and resolved spontaneously within 2 days. Fever (*n* = 1, 3.1%) occurred in one subject at the first treatment period following inhalation of the reference product, which was deemed unrelated to the drug and moderate; the subject recovered within 1 week after receiving a single 0.2 mg ibuprofen tablet.

No serious adverse events (SAEs) and deaths occurred during the study. Compared with baseline no clinically significant changes were found in vital signs, physical examinations, ECG results or laboratory findings. All AEs were reported to the Ethics Committee of Wuxi People's Hospital.

## 4 Dicussion

The bioequivalence of budesonide pMDI and Budiair was first reported in healthy Chinese subjects in our study. Both the test/reference GMR for *C*_max_ and 90% CIs of GMRs for AUC_0−*t*_ and AUC_0–∞_ were all within 80.00%–125.00%, confirming that the bioequivalence of the two products; additionally, both products also exhibited a favorable safety profile. Although this bioequivalence study was used to evaluate PK characteristics and systemic safety of budesonide in accordance with NMPA guidance [13], several key factors should also be considered in the design.

First of all, the crossover design is the most robust and valid method for comparing the PK profiles of test and reference products. It eliminates the inter‐subject variability in the comparison of average bioavailability and reduces the required sample size [18].

The reported within‐subject CVs for *C*_max_ of budesonide ranged from 50.0% to 92.2% [12]. High CV values reflect not only the drug's inherent variability but also variability arising from inhalation technique. On the basis of these CV values, the required sample size for a fully replicated crossover design would be approximately 68–184 subjects. Such huge sample sizes are not feasible in healthy volunteer studies because of ethical constraints and operational limitations. To minimize operational variability in drug administration, our study was conducted at an experienced inhaled‐product research center, and a CV of 30% has been proven to be adequate for powering the bioequivalence assessment [19]. Post hoc analysis using the observed parameters (CV = 44.26% and GMR = 0.97) with 80% power yielded a sample size of 38 subjects, closely matching the planned enrollment. The sample size of 32 subjects is statistically adequate and does not affect the bioequivalence conclusion.

Therefore, the four‐period crossover study conducted with 32 healthy subjects was methodologically appropriate, achieving optimal statistical power while minimizing the required sample size. In addition, in view of the short terminal elimination half‐life of budesonide [10], a 3‐day washout period was sufficient to eliminate the potential carryover effect.

In addition, healthy subjects are the most sensitive and suitable population for the bioequivalence study. Long‐term smoking impairs lung function, leading to airways and alveoli tissue damage, so smokers exhibit a higher risk of respiratory [20, 21]. Asthma patients often present with various complications and concomitant medications [22, 23], which may alter the PK profiles of budesonide. Conversely, healthy adults exhibit normal lung function and enhanced peripheral lung deposition, thereby increasing the sensitivity for detecting potential differences between test and reference products if present [24]. For this reason, the healthy, non‐smoking subjects were exclusively enrolled in our study.

What is more, the selection of the inhaled dose is crucial to the PK profiles of budesonide. Both FDA and NMPA guidelines for orally inhaled products (OIPs) emphasized that the minimum number of inhalations should be used to characterize the PK profiles [13, 14], 200 μg (200 μg/actuation × 1 actuation) is the recommended minimum single dose of Budiair. However, the extremely low plasma concentration of budesonide poses a significant challenge because of the poor systemic absorption. Thus, developing a sensitive analytical method is essential to accurately determine the PK profiles of budesonide. In order to minimize drug exposure in healthy subjects and accurately assess the PK differences between two products, a 200 μg dose of budesonide pMDI combined with the sensitive LC–MS/MS method was therefore adopted. This approach ensured that the PK profiles of budesonide in vivo could be accurately exhibited and distinguished at such a low dose. To our knowledge, this is the first study to reveal the PK profiles of 200 μg inhaled budesonide pMDI in healthy Chinese subjects, providing valuable insights for future research and regulatory evaluations.

Finally, proper use of the inhalation device is critical for subjects. Clinical studies have shown that over 77% of patients have experience in misusing inhaler devices, highlighting the importance of training and correct inhalation technique to ensure optimal therapeutic outcomes [25]. In our study, all subjects received standardized training before and after enrollment to achieve appropriate, consistent inspiratory flow rates and durations, which was also the requirement of the guidance [13, 26]. It should be noted that rigorous and individualized inhaler technique training is essential in all patient‐based clinical trials and is also the critical step to reduce operational deviations in pulmonary drug delivery. Only if patients correctly operate the inhalation device and strictly adhere to the prescribed regimen during long‐term therapy can the interindividual variability in pharmacodynamics be significantly reduced.

Notably, though the *t*_1/2_ of budesonide pMDI in our study was consistent with previously reported values, the median *T*_max_ was markedly shorter than before (0.03 h vs. 0.17–0.67 h) [10, 12, 27, 28]. Several factors may account for this discrepancy. First, the previous study used higher inhalation dosages [10, 12, 27, 28], which inherently required longer administration time; thereby the *T*_max_ was potentially prolonged. Second, the initial blood sampling timepoint (0.03 h post‐dose) was not designed in some PK studies because of the difficulty in sampling [28, 29]. Third, some PK data were derived from patient populations rather than healthy subjects [27, 30]. Additionally, variations in aerosol particle properties (size, distribution, morphology, etc.) were also determinants of pulmonary deposition and absorption rate of OIPs [31, 32], further influencing the PK parameters and systemic drug concentration.

Regarding the safety of budesonide, four subjects experienced at least 1 AE in our study. All AEs were mild to moderate and eventually resolved; no SAEs and deaths were reported. Both the test and reference products demonstrated excellent tolerability in healthy Chinese subjects after four single‐dose administrations. However, the limited sample size restricts adequate safety profile comparison between the test and reference products in healthy subjects. Thus, a comprehensive safety comparison of these two budesonides requires further evaluation in a larger‐scale patient trial with a long‐term dosing regimen.

## 5 Conclusion

The test product of budesonide pMDI (200 μg/actuation × 1 actuation) demonstrated the reference product (Budiair) and exhibited a favorable safety profile with good tolerability in healthy Chinese subjects.

## Author Contributions

Kai Huang, Shaohong Yin, and Weiguo Huang designed this clinical trial. Kai Huang, Wei Qin, Zhenzhong Qian, Ying Ding, Linling Que, and Yunfei Shi performed this clinical trial. Lairong Ding and Jinxia Gao analyzed and interpreted the data. All authors wrote, reviewed, and approved the final manuscript.

## Conflicts of Interest

Shaohong Yin, Weiguo Huang, Lairong Ding, and Jinxia Gao are employees of Lunan Better Pharmaceutical Co. LTD. Other authors declare no conflicts of interest.

## Data Availability

The data that support the findings of this study are available on request from the corresponding author.
